# Meckel's diverticulum complicated by a congenital intestinal adhesive band: a case report and literature review

**DOI:** 10.3389/fped.2026.1794967

**Published:** 2026-05-13

**Authors:** Yuming Wang, Tingliang Fu, Shuai Sun, Yewen Wang, Xiaoliang Xu, Lei Geng

**Affiliations:** Department of Pediatric Surgery, Binzhou Medical University Hospital, Binzhou, China

**Keywords:** children, comorbidity, gangrene, ileal perforation, Meckel's diverticulum, surgical management

## Abstract

As one of the most common congenital gastrointestinal malformations, Meckel's diverticulum (MD) often has its clinical significance underestimated due to its atypical manifestations. MD may coexist as “concomitant lesions” with various abdominal surgical diseases, increasing the risk of misdiagnosis and missed diagnosis. This article reports an extremely rare case of a 5-month-old male infant with necrotic Meckel's diverticulitis coexisting terminal ileal necrosis and perforation due to a congenital intraabdominal fibrous band. Through a systematic review of the literature on MD with surgical complications associated with other surgical diseases in the PubMed database from 1982 onward (including a total of 12 cases), this study aims to explore the diagnostic challenges and treatment strategies of this condition.

## Introduction

Meckel's diverticulum (MD) is one of the most common congenital gastrointestinal malformations, resulting from the incomplete obliteration of the vitelline duct (omphalomesenteric duct) during embryonic development (1–3). It has a prevalence of approximately 2% in the general population, is more common in males than females, and is typically located on the antimesenteric border of the ileum, approximately 60–100 cm proximal to the ileocecal valve (2, 4). The majority of affected individuals remain asymptomatic throughout their lives, with only about 4%–6% developing complications, which occur predominantly in children, especially in those under two years of age (2, 5). Due to its atypical clinical presentation, MD is frequently confused with other acute abdominal conditions, such as acute appendicitis or intussusception, posing challenges for preoperative diagnosis (1). Imaging modalities, such as ultrasonography(US), computed tomography (CT), and ⁹⁹mTc-pertechnetate scintigraphy, can serve as auxiliary diagnostic tools. However, definitive diagnosis often relies on intraoperative exploration via laparotomy or laparoscopy for suspected appendicitis (1). The coexistence of MD with other surgical diseases is even rarer. These diseases may include congenital malformations, e.g., intestinal malrotation, duplication cyst, or other surgical emergencies, such as appendicitis. Herein, we report an extremely rare case of an infant with gangrene MD coexisting ileal necrosis due to a congenital fibrous band compression, which was successfully managed in the mean time.

## Case report

The patient was a male infant, aged 5 months and 13 days, weighing 8.0 kg.

**Figure 1:**
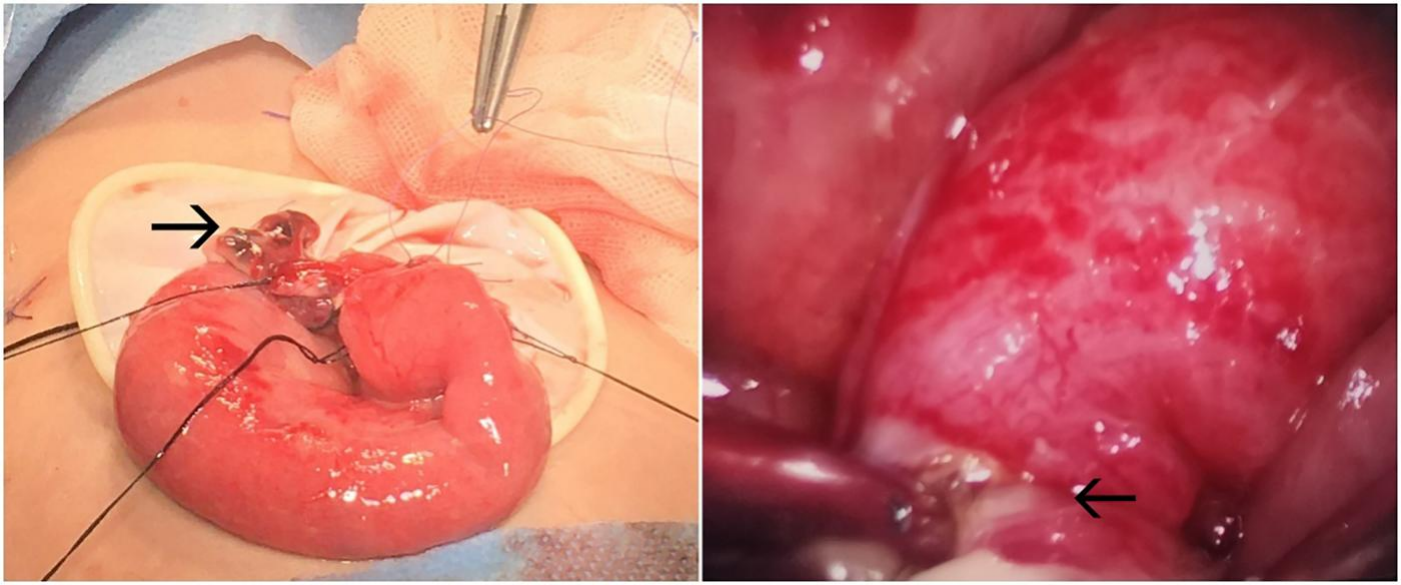
Surgical photograph. The arrow on the left indicates a perforated and necrotic diverticulum; the arrow on the right points to a constricting band causing intestinal entrapment.

The infant was admitted to the Pediatric Gastroenterology Department due to a 3-day history of fever and vomiting. His body temperature was 38℃, heart rate 120 beats per minute, respiratory rate 30 breaths per minute, oxygen saturation 96%. The vomitus consisted of gastric contents without bile or coffee-ground material. Sunken eyes and slightly distended abdomen. The initial diagnoses were enteritis, infectious fever with moderate dehydration. Intravenous access was immediately established. Intravenous antibiotics (ceftriaxone) were administered for infection, intravenous fluid replacement was given to correct dehydration, and proton pump inhibitor (omeprazole) was used to protect the gastric mucosa. Blood tests revealed that white blood cell count of 3.1 × 10⁹/L, red blood cell count of 4.0 × 10^12^/L, hemoglobin of 107 g/L, platelet count of 347 × 10⁹/L, neutrophil percentage of 50.6%, erythrocyte sedimentation rate（ESR） of 38 mm/h, procalcitonin of 3.01 ng/mL, B-type natriuretic peptide precursor of 10.00 pg/mL, C-reactive protein of 186.30 mg/L, serum lipase of 25.6 U/L, amylase of 10.9 U/L, weakly positive mycoplasma pneumoniae, and no abnormalities in liver function, myocardial enzymes, or hepatitis B serology. An upright abdominal x-ray indicated: partial intestinal distension and gas accumulation in the abdomen, with multiple air-fluid levels, some arranged in a step-like pattern. No definite free air was observed under the bilateral diaphragms, suggesting intestinal obstruction. Repeat physical examination revealed abdominal distension, increased abdominal wall tension, and the infant cried incessantly upon palpation. Small intestinal obstruction with diffuse peritonitis was considered, along with systemic infection and fluid-electrolyte imbalance. The American Society of Anesthesiologists Physical Status Classification (ASA) was grade IV. After transfer to the Pediatric Surgery Department, immediate surgical intervention was performed. Diagnostic laparoscopy was performed. Intraoperatively, diffuse distension of the intestinal loops was observed, with some segments showing congestion and edema. Diffuse purulent fluid was present in the abdominal cavity, along with intestinal adhesions and abscess formation between small intestinal loops. A laparoscopic grasper was inserted. Intestinal adhesions were lysed using blunt dissection combined with an electrosurgical hook (coagulation mode, 20 W), and the abscess was evacuated. A cord-like band tightly compressed the terminal ileum. A silk suture was used to lift the band and then transect and release the band via electrocoagulation hook, revealing necrosis and perforation of the terminal ileal wall. Approximately 25 cm from the ileocecal junction, a gangrene Meckel's diverticulum with abscess formation was found. The purulent fluid and leaked intestinal fluid were completely aspirated. A silk suture was used to ligate the perforated diverticulum. The umbilical incision was extended, and the intestinal loop was exteriorized for a wedge resection of the MD with primary intestinal anastomosis, along with resection and anastomosis of the necrotic and perforated terminal ileal wall. Prior to anastomosis, the intestinal contents were appropriately aspirated to decompress and relieve abdominal distension (Figure 1).

The intraoperative diagnoses were: 1. Meckel's diverticulum necrosis and perforation; 2. Terminal ileum strangulation necrosis and perforation; 3. Intestinal obstruction caused by congenital band.Histopathological examination of the resected specimens revealed: (Meckel's diverticulum) The examined intestinal wall tissue showed predominantly hemorrhagic infarction with inflammatory exudate. (Necrotic terminal ileal wall) The examined intestinal wall tissue exhibited full-thickness vascular dilation and congestion, along with a significant amount of necrotic tissue. On the first postoperative day, a repeat blood test, erythrocyte sedimentation rate, and reticulocyte count showed: white blood cell count of 4.8 × 10⁹/L, red blood cell count of 2.8 × 10^12^/L, hemoglobin of 71 g/L, platelet count of 192 × 10⁹/L, neutrophil percentage of 68.9%, ESR of 2.0 mm/h, procalcitonin of 28.37 ng/mL, and reticulocyte percentage of 1.8%. Postoperatively, the patient received meropenem at a dose of 0.48 g/day for 8 days and metronidazole at a dose of 0.18 g/day for 10 days. Gastrointestinal decompression and parenteral nutrition support were provided. On postoperative day 2, the infant's crying decreased. By postoperative day 3, after starting enteral feeding, there was no vomiting or abdominal distension, and the abdominal pain had completely resolved. The feeding amount was then gradually increased. On the 15th postoperative day, a repeat hematological examination showed no significant abnormalities. The patient was hospitalized for 16 days postoperatively. During a one-year follow-up, the child exhibited normal growth and development without recurrence of intestinal obstruction or other discomforting symptoms.

## Discussion

A computerized search of the PubMed database was performed to identify relevant literature published from 1982 to the present, using the keywords Meckel's diverticulum, surgical treatment, small intestinal diverticulum, comorbidity, gastrointestinal malformation, and gastrointestinal bleeding. We reviewed 11 articles and the case reported in this paper, identifying a total of 12 cases of Meckel's diverticulum coexisting with other diseases (6–16). We synthesized the cases reported in these articles by evaluating their clinical presentations, comorbidities, diagnoses, pathological findings, and treatments (Table 1).

**Table 1:** Demographics, clinical features, and outcomes in complicated Meckel's diverticulum with coexisting surgical disorders.

Reference	Sex	Age	Symptomatolgy	Initial Diagnosis	Definitive Diagnosis	Comorbidities	Operative Findings	Pathological Findings	Surgical Management	Outcomes
Jacob F. Quail, 2014 (6)	M	12 Y	Abdominal Pain, Groin pain	Acute Appendicitis	Appendicitis Associated with a Strangulated Littre's Hernia	Acute Appendicitis	Gangrenous MD without Gross Perforation	Hemorrhagic Fibrovascular Tissue (MD); Early Acute Appendicitis	Diverticulectomy and Appendectomy	A
Darko Katalinic, 2014 (7)	M	63 Y	Abdominal Distension or Diarrhoea	Colon Adenocarcinoma	Neuroendocrine Tumor Arising in MD Coexisting with Colon Adenocarcinoma	Colon Cancer	Colonic Tumor Coexisting with a Mass in MD	Poorly Differentiated Colon Adenocarcinoma with Liver Metastases; Neuroendocrine Tumor in MD	Right Hemicolectomy with Ileotransverse Anastomosis	A
Henry Taylor, 201 5(8)	M	8 Y	Abdominal Pain,Vomiting, Fever	Viral Gastroenteritis or Mesen- teric Adenitis	MD with Intestinal Malrotation	Intestinal Malrotation	Long, Gangrenous, Perforated MD,Around this MD,the small bowel had torted 360°	Perforated MD Forming Abscess Wall	Diverticulectomy and Ladd's Procedure	A
N. Mudatsakis, 2011 (9)	M	42 Y	Abdominal Pain, Vomiting, Anorexia, Nausea	Acute Appendicitis	Acute Appendicitis with Carcinoid Tumor in MD	Acute Appendicitis,Carcinoid Tumor	MD Located 60 cm Proximal to Ileocecal Valve	Carcinoid (<1 cm) in MD, Extending to Serosa, Intact Mucosa	Diverticulectomy and Appendectomy	A
Prakash Rau, 1982 (10)	F	28 Y	Cramping Lower Abdominal Pain; Bloody Diarrhea	IBD	Bleeding MD with IBD	Crohn's Disease	Ulcerated MD (2 ft from ICV) with Distal Blood Accumulation	MD with Pyloric-type Epithelium and Chronic Inflammation	Diverticulectomy	A
Turyalai Hakimi, 2025 (11)	M	15 MO	Bloody Diarrhea, and Abdominal Distension, Abdominal Pain, Fever, Shock, Dehydration	Intussusception	Cecal Duplication Cyst and MD Causing Intussusception	Cecal Duplication Cyst	Cecal Duplication Cyst and a Walnut-sized MD	Duplication Cyst	Right hemicolectomy, Diverticulectomy and end-to-end anastomosis, End-to-end ileotransverse anastomosis	A
A. L. Gidwani, 2008 (12)	M	51 Y	Abdominal Pain,Diarrhea	NA	Acute Appendicitis, Crohn's Disease, Non-inflamed MD with Carcinoid Tumor	Acute Appendicitis, Crohn's Disease,Carcinoid Tumor	Perforated Appendix, Thickened Terminal Ileum (suggestive of Crohn's), and Adjacent Broad-based MD	Perforated Appendicitis; Incidental Benign Carcinoid (<0.5 cm)	Appendectomy	A
Islam H. Metwally, 2016, (13)	M	49 Y	Pelvic Pain, Frequent Micturition	Neurogenic Tumor	GIST Arising from MD	GIST	Ileal Mass at MD Tip with Cystic Changes	High-risk GIST Arising from MD	Diverticulectomy	A
Faiza Azeema Shaikh, 2024 (14)	F	4 Y	fever, vomiting, abdominal pain, and abdominal disten- sion	Appendicular Perforation	Perforated MD with Acute Appendicitis	Acute Appendicitis	Perforated MD and Acute Appendix	MD (4 × 3 cm, broad-based) with Congestion, Necrosis, Ileal-type Mucosa	Diverticulectomy and Appendectomy	A
Cristina Æerban, 2020 (15)	NA	71 Y	Abdominal Pain,bilious vomiting	NA	Perforated GIST and MD	GIST	Perforated Ileal Tumor and MD	Malignant GIST (Fibrosarcoma-like) Involving Full Intestinal Wall	Segmental enterectomy, enteroenterostomy, and peritoneal drainage.	D
Harveen K Lamba, 2016 (16)	F	64 Y	Nausea, Bilious Emesis, Post-Prandial Abdominal Pain, Tachypneic	Cholecystoduodenal fistula complicated by gallstone ileus.	Gallstone ileus associated with impaction at MD	Gallstone Ileus	Large Gallstone Obstructing Jejunum; MD Impacted with Smaller Stones	Ulceration and Transmural Inflammation in Resected Bowel; Mixed Gallstones Found	Partial Small Bowel Resection and End-to-end Anastomosis	A
The present case	M	5 MO 13 Days	Fever, Vomiting	Pediatric Enteritis; Infectious Fever; Moderate Dehydration	MD complicated by adhesive intestinal band formation	Adhesive Intestinal Band Formation	Internal Hernia (Adhesive Band) with Necrotic and Perforated MD	The MD showed extensive hemorrhagic infarction and inflammatory exudate, while the terminal ileal wall displayed full-thickness vascular dilation, congestion, and abundant necrotic tissue.	Resection of Meckel's diverticulum and the necrotic terminal ileum with primary anastomosis	A

M, male; F, female; Y, years; MO, months; A, alive; D, death; NA, not available; MD, Meckel's diverticulum; IBD, inflammatory bowel disease; GIST, gastro-intestinal stromal tumor; ICV, ileocecal valve.

MD occurs in 2% of the population, and only 2% of individuals with MD become symptomatic (2, 14, 17–21). Its clinical significance extends far beyond its own pathological changes. More noteworthy is that MD often acts as a “comorbid condition” or “underlying cause,” coexisting with various abdominal diseases to form complex and easily misdiagnosed clinical syndromes. The clinical manifestations of symptomatic MD are diverse, primarily related to complications and associated diseases, often presenting as gastrointestinal bleeding, mechanical intestinal obstruction, non-specific abdominal pain, and acute inflammation (8, 10, 22).

In this case series, patients presented with abdominal pain (in 8 cases), primarily localized around the periumbilical region, right iliac fossa, or lower abdomen. Other symptoms included fever (in 4 cases), hematochezia (in 2 cases), nausea and vomiting (in 5 cases), abdominal distension or diarrhea (in 4 cases), dehydration (in 1 case), and even shock (in 1 case).

Symptomatic MD shares similar clinical manifestations with acute appendicitis, with the incidence of acute appendicitis being approximately 50 times higher than that of symptomatic MD (17, 20, 23). Among this case series, four patients presented with MD complicated by acute appendicitis. Of these, three underwent diverticulectomy and appendectomy, while one received appendectomy alone. It is recommended to routinely search for the presence of MD during appendectomy; however, the management of incidentally discovered and asymptomatic MD remains controversial (5, 12). Most surgeons would advocate for the resection of incidentally discovered asymptomatic diverticula in pediatric patients to mitigate the risk of secondary complications (12, 13, 16, 18, 24). In contrast, the literature provides less conclusive guidance regarding the prophylactic resection of asymptomatic MD in adults. An analysis of 1,476 cases from the Mayo Clinic did not explicitly support the routine resection of incidental MD but suggested its selective removal in the presence of factors associated with MD-related complications, such as age under 50 years, male sex, diverticulum length greater than 2 cm, and the presence of heterotopic tissue (24). There is a broader consensus that complicated and symptomatic MD requires surgical intervention (13, 14, 25).

MD may coexist with other surgical diseases. This case series includes one patient with an associated cecal duplication cyst that triggered intussusception, and another with intestinal malrotation leading to torsion and perforation of the MD. Both scenarios suggest that during embryonic development, the persistence of the vitelline duct (MD) and abnormalities in intestinal rotation/fixation or duplication may share a common embryological disturbance (26). In pediatric patients, since cecal duplication cysts, intestinal malrotation, and MD can all present as acute abdominal conditions, such as intestinal obstruction, the treatment of these coexisting anomalies requires combined management. As demonstrated in this series, this involved simultaneous diverticulectomy along with Ladd's procedure or resection of the duplication cyst (11). In our patient, the MD was necrotic and perforated, and was associated with a congenital intraabdominal fibrotic band. This band tightly compressed the terminal ileum, resulting in acute intestinal obstruction and necrosis of the bowel wall. The management consisted of diverticulectomy, resection of the necrotic terminal ileum, and primary anastomosis.

MD can be a site of involvement for Crohn's disease, and its ulceration, bleeding, or obstructive symptoms may mask or confuse the typical presentation of inflammatory bowel disease (IBD). In this series, 2 patients had coexisting MD and Crohn's disease. For patients suspected or confirmed to have IBD, if their symptoms, imaging, or endoscopic findings are atypical, the possibility of coexisting MD should be considered (10). Treatment should address both conditions; resection of symptomatic or complicated MD may help alleviate local symptoms but should be conducted within the overall treatment framework for IBD (12). Reports of MD masquerading as Crohn's disease are rare, but MD diagnosed in patients with Crohn's disease is not uncommon. However, it remains unclear whether the prevalence of Meckel's diverticulum is increased in patients diagnosed with Crohn's disease (10, 27).

The clinical manifestations of symptomatic MD are not uniform but are closely related to the underlying pathological process. Bleeding is usually caused by ulceration of ectopic gastric mucosa within the diverticulum. If diverticulitis occurs, it may progress to perforation and peritonitis (5). Obstruction may result from intussusception secondary to MD, and torsion of MD has also been reported. Furthermore, MD can be associated with various benign or malignant tumors, as well as the less common Littre hernia (6, 13, 28). However, the symptoms of MD are often vague and overlap considerably with those of other acute abdominal conditions, making diagnosis difficult. Symptomatic MD is more common in children, especially male infants, primarily presenting as bleeding from ectopic gastric mucosa or obstruction. Symptomatic MD is rare in adults; when it does occur, diverticulitis and obstruction are the most common presentations (1, 5). Perforation of MD is relatively uncommon and usually results from inflammation and necrosis caused by a stone obstructing the diverticulum (16). Of note, the presence of ectopic mucosa in MD can predispose to several types of tumors, and the risk of neoplasm in patients with MD is higher than in any other part of the small intestine, with carcinoid being the most common, accounting for two-thirds of MD-associated tumors (25, 29). In patients with suspected appendicitis undergoing laparoscopy in whom the appendix appears normal, surgeons should actively explore for the presence of an inflamed MD. When the diagnosis is in doubt, diagnostic laparoscopy is of great clinical value (30). In our case, diagnostic laparoscopic exploration not only confirmed necrotic perforation of MD but also revealed an occult congenital intestinal fibrous band causing obstruction, thereby avoiding a missed life-threatening coexisting lesion. Therefore, for patients with symptomatic MD, especially those with an unclear preoperative diagnosis or presenting with intestinal obstruction or peritonitis, diagnostic laparoscopy should be the preferred initial surgical approach, allowing systematic exploration of the entire abdominal cavity to avoid missing rare or occult diseases (31).

## Conclusion

Symptomatic Meckel's diverticulum (MD) often coexists as a “comorbid condition” with various congenital anomalies, tumors, inflammatory bowel disease, or acute abdominal conditions, constituting a complex clinical syndrome. Therefore, when symptomatic MD is confirmed intraoperatively, surgeons should regard it as an important warning sign to explore other potential intra-abdominal lesions. After resection of MD, a routine and systematic exploration of the entire abdominal cavity should be performed to avoid missing life-threatening coexisting diseases. Diagnostic laparoscopy, with its advantages of panoramic abdominal visualization, minimal invasiveness, precision, and flexible decision-making, should be the preferred initial surgical approach for patients with symptomatic MD who have an unclear preoperative diagnosis or present with intestinal obstruction or peritonitis. Enhancing the awareness of coexisting diseases associated with MD and standardizing the intraoperative exploration process are of great clinical significance for timely diagnosis, appropriate treatment, and improvement of patient outcomes.

## Data Availability

The original contributions presented in the study are included in the article/Supplementary Material, further inquiries can be directed to the corresponding authors.
